# Supplementary material for: Impact of annual community-directed treatment with ivermectin on the incidence of epilepsy in Mvolo, a two-year prospective study
Source: PLoS Negl Trop Dis. 2024 Mar 21;18(3):e0012059. doi: 10.1371/journal.pntd.0012059 (PMC10986994; doi:10.1371/journal.pntd.0012059)
Supplement: S1 Material — Questionnaire A–Epilepsy screening questionnaire delivered to every element of each household to identify persons with suspected epilepsy. Questionnaire B–Neurology questionnaire delivered to the persons with suspected epilepsy to confirm or reject the epilepsy diagnosis. Fig A–Incidence of epilepsy trends over time in Mvolo County. Table A–Gender-specific epilepsy prevalence in 2020 and 2022 in Mvolo County. Table B—Obvious causes of epilepsy. Table C–Longitudinal decrease in the incidence of overall epilepsy and probable nodding syndrome (NS) after reintroducing ivermectin distribution in Maridi and Mvolo Counties. (PDF) [file pntd.0012059.s007.pdf]

Supplementary Word document of "Impact of annual community-directed treatment with ivermectin on the incidence of epilepsy in Mvolo, a two-year prospective study"

Short title: Epilepsy in an onchocerciasis-endemic county under ivermectin

Luís-Jorge Amaral[1*†], Stephen Raimon Jada[2*], Aimee Kemayou Ndjanfa[1], Jane Y. Carter[3], Gasim Abd-Elfarag[2, 4, 5], Samuel Okaro[2], Makoy Yibi Logora[6], Yak Yak Bol[6], Thomson Lakwo[7], Joseph N Siewe Fodjo[1] and Robert Colebunders[1]

[1] Global Health Institute, University of Antwerp, 2610 Antwerp, Belgium.
[2] Amref Health Africa, P.O. Box 410 Juba, South Sudan.
[3] Amref Health Africa Headquarters, P. O. Box 27691 – 00506, Nairobi, Kenya.
[4] Access for Humanity, 647 Hai Gudele Block 7, Juba, South Sudan.
[5] School of Public Health, University of Juba, Juba, South Sudan.
[6] Neglected Tropical Diseases Unit, Ministry of Health, P.O. Box 410 Juba, South Sudan.
[7] Vector Control Division, Ministry of Health, Kampala, Uganda.
* Contributed equally
† Correspondence: luis-jorge.telesdemenesesdoamaral@uantwerpen.be
Current address: Global Health Institute, University of Antwerp, Kinsbergen Centrum, Doornstraat 331, 2610 Antwerp, Belgium.

Table of contents

Questionnaire A – Epilepsy screening questionnaire delivered to every element of each household to identify persons with suspected epilepsy 2

Questionnaire B – Neurology questionnaire delivered to the persons with suspected epilepsy to confirm or reject the epilepsy diagnosis 3

Figure A – Incidence of epilepsy trends over time in Mvolo County 9

Table A – Gender-specific epilepsy prevalence in 2020 and 2022 in Mvolo County 10

Table B — Obvious causes of epilepsy 11

Table C – Longitudinal decrease in the incidence of overall epilepsy and probable nodding syndrome (NS) after reintroducing ivermectin distribution in Maridi and Mvolo Counties 12

Questionnaire A – Epilepsy screening questionnaire delivered to every element of each household to identify persons with suspected epilepsy

HOUSEHOLD SURVEY QUESTIONNAIRE: SCREENING FOR EPILEPSY

Investigator ID: ____________________ Signature:____________________

Village: ____________________ Date current visit (DD/MM/YYYY): __ __/__ __/__ __ __ __

Previous HOUSEHOLD CODE __ __ __/__ __/__ __ __ Date previous visit __ __/__ __/__ __ __ __ . GPS________________

1) Household Head : ________________________________ Etnicity________________

2) Does family originate from this villag ☐ YES ☐ NO If NO, How long have they been residing in this village? ___________ (years)

3) Main income generating activity of the famil

Farming cattle pigs fishing shop employe soldier other, specify____________________

4) Has anybody developed died in the household since last survey?

If YES, Name : ______age ___________sex______When _____month_____year

4) Has anybody developed epilepsy/nodding in the household since last survey?

If YES, Name : ____age ___________sex______When _____month_____year

1. No. pers.		2. Age	3. Gender	4.1 QUESTION 1			4.2 QUESTION 2			4.3 QUESTION 3			4.4 QUESTION 4			4.5 QUESTION 5			4.6 Itching			4.7 Blind	4.8 IVM
Pst	Abs	Year: Y Month: M	M=Male F=Female	Loss of consciousness with either urine on self and/or drooling?			Absence(s) or loss of contact with the surrounding of sudden onset and brief duration?			Jerking or uncontrolled abnormal movement (convulsion) of the limb(s) of sudden onset and lasting for a few minutes?			Head nodding?			Has it ever been said that he/she is epileptic or has he already had 2 or more seizures?						1 or 2 eye	IVM in 2019?
1 ☐	☐		M ☐ F ☐	YES ☐	NO ☐	? ☐	YES ☐	NO ☐	? ☐	YES ☐	NO ☐	? ☐	YES ☐	NO ☐	? ☐	YES ☐	NO ☐	? ☐	YES ☐	NO ☐	? ☐		
2 ☐	☐		M ☐ F ☐	YES ☐	NO ☐	? ☐	YES ☐	NO ☐	? ☐	YES ☐	NO ☐	? ☐	YES ☐	NO ☐	? ☐	YES ☐	NO ☐	? ☐	YES ☐	NO ☐	? ☐		

PERSONS SUSPECTED TO HAVE EPILEPSY (REFER TO DOCTOR / NEUROLOGIST) :

CODE (I/VV/HHH/N°): __ __ __/__ __/__ __ __/__ __ Name: ____________________ Sex (M/F): ____ Age: _______
CODE (I/VV/HHH/N°): __ __ __/__ __/__ __ __/__ __ Name: ____________________ Sex (M/F): ____ Age: _______
CODE (I/VV/HHH/N°): __ __ __/__ __/__ __ __/__ __ Name: ____________________ Sex (M/F): ____ Age: _______
CODE (I/VV/HHH/N°): __ __ __/__ __/__ __ __/__ __ Name: ____________________ Sex (M/F): ____ Age: _______

*Ethnicity was only asked in the first survey.

Questionnaire B – Neurology questionnaire delivered to the persons with suspected epilepsy to confirmor reject the epilepsy diagnosis

Neurology questionnaire (stage 2)

DATE : _____/_____/ ________

PARTICIPANT IDENTIFICATION

1. Participant ID: Number AS (3 capital letters) number village (2 capital letters) number household (3 digits) number family member (2 digits)
2. First Name:____
3. Surname Name: ____
4. Age years
5. Gender ☐ male ☐ Female
6. Ethnic group:____
7. Was the person living in the village since birth ☐YES ☐NO ☐DON'T KNOW
8. If not, since how long does the person live in the village ________years (if less than 1year put 0)
9. What is the interviewed participant's duration of stay in the survey area__________years

HISTORY OF EPILEPSY

SEIZURE TYPOLOGY

10. Has the participant ever suddenly lost consciousness ☐YES ☐NO ☐DON'T KNOW

    If yes, did s/he experience any of the following?

    i. Loss of bladder control? ☐YES ☐NO ☐DON'T KNOW
    ii. Foam at the mouth? ☐YES ☐NO ☐DON'T KNOW
    iii. Biting of the tongue? ☐YES ☐NO ☐DON'T KNOW
11. Has the participant ever experienced absence(s) or sudden loss(es) of contact with the surroundings, for a short duration of time? ☐YES ☐NO ☐DON'T KNOW
12. Does the participant have a history of head nodding?

    ☐ YES, in the past ☐ YES, still ongoing ☐ NO ☐ DON'T KNOW

    IF YES, specify age of the participant at onset of head nodding_______years

    If the nodding stopped, at what age did it stop? _____________years
13. Has the participant ever experienced sudden, uncontrollable twitching or shaking of arms, legs or head, for a period of a few minutes with amnesia (deficit of memory)? ☐YES ☐NO ☐DON'T KNOW
14. Does the participant sometimes experience sudden and brief bodily sensations, see or hear things that are not there, or smell strange odours? ☐YES ☐NO ☐DON'T KNOW
15. Has the participant ever been told that he / she is suffering from epilepsy or that he / she has had epileptic fits? ☐YES ☐NO ☐DON'T KNOW

If so was the diagnosis confirmed by a medical doctor? ☐YES ☐NO ☐DON'T KNOW?

16. What type are currently the most frequent seizures (more than one answer possible)
    - ☐ Generalised convulsive seizures
    - ☐ Atonic seizures (drop attacks)
    - ☐ Absences
    - ☐ Nodding seizures
    - ☐ Focal motoric seizures without loss of consciousness
    - ☐ Focal motoric seizures with decreased consciousness
    - ☐ One seizure
    - ☐ Others, specify:_______________________________
    - ☐ No seizure. If no, skip to General examinations
17. At what age did the seizures start?_____year ☐ DON'T KNOW (999) ☐ NA (Not applicable)(888)
18. Did the seizures start less than one year ago? ☐ YES ☐ NO ☐ DON'T KNOW
19. If yes, since how many months? ___________________Months
20. What triggers the seizures / head nodding? (tick all that apply)
    - ☐Spontaneous (no obvious trigger)
    - ☐Sight of food ☐Cold weather
    - ☐DON'T KNOW
    - ☐ Other, specify_________________________

SEIZURE HISTORY

21. What is the number of epileptic seizures since onset? ☐ Two ☐ Three or more seizures

    If only two seizures, were they more than 24h apart? ☐ YES ☐ NO ☐ DON'T KNOW ☐ NA
22. Has the participant had a seizure in the last 5 years? ☐ YES ☐ NO ☐ DON'T KNOW
23. Has the participant had a seizure in the last 12 months? ☐ YES ☐ NO ☐ DON'T KNOW
24. What is the current frequency of the seizures?
    - ☐ Every day
    - ☐ Every week
    - ☐ Every month
    - ☐ Every year

    Specify number: ______________
25. a. How many seizures did you have LAST WEEK?

    ☐ None ☐ 1-4 episodes ☐ More than 4 ☐ DON'T KNOW

    b. What is the average duration of a seizure episode?

    ☐ less than a minute ☐ 1-5 minutes ☐ more than 5 minutes ☐ DON'T KNOW

MEDICAL HISTORY

26. Family history of seizures ☐ YES ☐ NO ☐ DON'T KNOW

    IF YES, specify who these are (tick all that apply)
    - ☐Siblings (brother/sister); No. of affected siblings _______
    - ☐Father ☐Mother ☐Grandparent(s)

☐ Other, Specify ________________________________

Pregnancy and Birth:

27. Did the pregnancy of the mother of the participant proceed normally? ☐ YES ☐ NO ☐ DON'T KNOW

If NO, specify:_________________________________ ____

28. Was the participant born at term (pregnancy had completed 9 months)? ☐ YES ☐ NO ☐ DON'T KNOW

29. Was there a delayed cry at birth? ☐ YES ☐ NO ☐ DON'T KNOW

Psychomotor Development during Childhood:

Prior to onset of seizures

30. Was the child growing normally prior to the onset of the seizures? ☐ Yes ☐ No ☐ DON'T KNOW

IF NO, at what age did the abnormal growing appear?_______years

31. Did the child learn to do things like other children of his/her age prior to the onset of the seizures?

☐ Yes ☐ No ☐ DON'T KNOW

IF NO, at what age did the learning difficulty start?_______year

32. Compared with other children of his/her age, did the child appear in any way mentally backward, dull or slow before the onset of the seizures? ☐ Yes ☐ No ☐ DON'T KNOW

IF YES, at what age did it start?_______years

Occurrence of severe disease in the past:

33. Has the interviewed participant suffered from severe measles preceding the onset of epileptic seizures? ☐ YES ☐ NO ☐ DON'T KNOW

If yes, how long before the onset of seizures..............................Years

34. Has the interviewed participant suffered from a severe form of malaria (hospitalised) preceding the onset of epileptic seizures? ☐ YES ☐ NO ☐ DON'T KNOW

If yes, how long before the onset of seizures..............................Years

35. Has the interviewed participant suffered from encephalitis/meningitis preceding the onset of epileptic seizures? ☐ YES ☐ NO ☐ DON'T KNOW

If yes, how long before the onset of seizures .... Years

36. Has the participant had a head injury with loss of consciousness preceding the onset of epileptic seizures? ☐ YES ☐ NO ☐ DON'T KNOW

If yes, how long before the onset of seizures .... Years

37. Has the participant had a prolonged posttraumatic coma before the onset of epileptic seizures?

☐ YES ☐ NO ☐ DON'T KNOW

If yes, how long before the onset of seizures .... Years

38. Was the onset of epilepsy following another illness?

☐ YES ☐ NO ☐ DON'T KNOW

If YES, specify the illness ____

39. **PHYSICAL EXAMINATION Done** ☐ YES ☐ NO if no go to 61

40. Vision ☐ Normal ☐ Reduced ☐ BLIND, one eye affected ☐ BLIND, both eyes affected

41. Thoracic/spinal abnormalities ☐ YES ☐ NO ☐ DON'T KNOW

IF YES, specify________________________________ ____________________________

42. Facial abnormalities ☐ YES ☐ NO

IF YES, specify ____

43. Does the adolescent (> 16 years old) /adult looks like a child? ☐ YES ☐ NO ☐ NA

44. If yes, external signs of sexual development conform to age:

☐ YES ☐ NO ☐ EXAMINATION DECLINED ☐ NA

If NO, specify:

45. girls: ☐ breast not developed ☐ NA

46. girls and boys: ☐ no pubic hear ☐ NA

47. Cervical Lymph nodes ☐ YES ☐ NO

48. Onchocerciasis Nodules ☐ YES ☐ NO

49. Itching ☐ YES ☐ NO

50. Burn lesions ☐ YES ☐ NO

51. Dermatological examination (several answers possible) ☐ Normal ☐ papular/nodular pruritic skin ☐ leopard skin ☐ dry, thickened, wrinkled skin ☐ other skin abnormality

Other dermatological lesion (specify) __________________________

NEUROLOGICAL EXAMINATION

52. Is the participant alert? ☐ YES ☐ NO

53. Fully oriented in place/time/person ☐ YES ☐ NO

54. Is the participant's mentally retarded? ☐ YES ☐ NO

55. Generalised muscle wasting ☐ YES ☐ NO

56. Paresis ☐ YES ☐ NO

57. Is the participant walking normally? ☐YES ☐ NO ☐ DON'T KNOW

58. If no specify_______________________________________

Psychiatric symptoms

59. Does the participant suffer from any behavioural problem? ☐ YES ☐ NO

IF YES, specify: ____

60. <u>Physical / Functional Indices</u>

Modified Rankin Scale: Please mark the most accurate description of the current functional state of the person with epilepsy, as observed during the evaluation

1	No significant disability despite symptoms; able to carry out all usual duties and activities
2	Slight disability; unable to carry out all previous activities, but able to look after own affairs without assistance
3	Moderate disability; requiring some help, but able to walk without assistance
4	Moderately severe disability; unable to walk without assistance and unable to attend to own bodily needs without assistance
5	Severe disability; bedridden, incontinent and requiring constant nursing care and attention

61. ***Epilepsy CASE CLASSIFICATION***

☐ Head nodding syndrome
☐ Head nodding syndrome plus (including other seizure types)
☐ Epilepsy without head nodding
☐ Other diagnosis

62. If NO epilepsy: other diagnosis?

☐ One seizure
☐ Recurrent febrile convulsions
☐ Dizziness / syncope
☐ Paroxysmal vertigo
☐ Alcohol/drug use
☐ Severe anaemia
☐ Severe protein malnutrition
☐ psychogenic non epileptic syndrome (PNES)
☐ Mental retardation without epilepsy
☐ Psychiatric illness without epilepsy
☐ Classic migraine
☐ Other, specify_______________________

ANTI-EPILEPTIC TREATMENT

63. What is or was the type of seizure medication taken by the participant?

☐ No treatment ☐ DON'T KNOW
☐ Traditional ☐ anti-epileptic drug
☐ Mixed ☐ NA

If No treatment or Traditional or Don't know or NA: Go to Ivermectin use (68)

64. *If anti-epileptic drug treatment:* Which substance is taken by the participant (more than one answer possible)
☐ Phenobarbital
☐ Sodium valproate
☐ Phenytoin
☐ Carbamazepine
☐ Other anti-epileptic

If other, specify: ______________________________

65. Is the participant taking the treatment regularly?
☐ currently (took it every day last week) ☐ only in the past ☐ DON'T KNOW

If only in the past, why?
☐ Personal reasons
☐ (Temporary) non-availability of medication
☐ Lack of financial means to buy medication
☐ DON'T KNOW
☐ Other, specify ____________________

IVERMECTIN USE

66. Has the participant ever received ivermectin? ☐ YES ☐ NO ☐ DON'T KNOW ☐ NA

IF YES: how many years the person took ivermectin__________years

Has the participant taken ivermectin in 2021?

☐ YES ☐ NO ☐ DON'T KNOW

Full physician name ____________________

Figure A – Incidence of epilepsy trends over time in Mvolo County

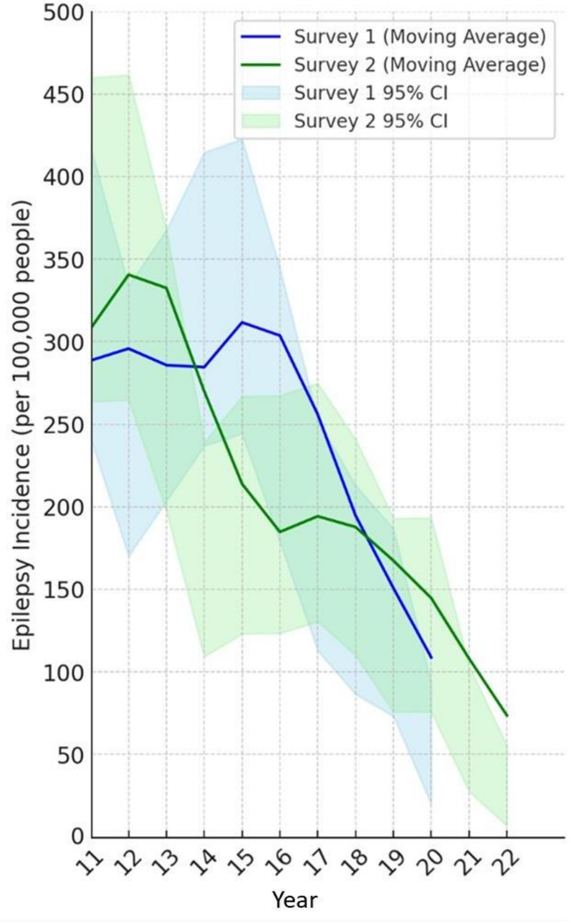

Figure A – Epilepsy incidence over time in Mvolo County, South Sudan, utilising 3-year moving averages to mitigate recall bias and reduce data heterogeneity. The 95% confidence intervals (CIs) for each annual incidence rate were presented, facilitating comparison with the moving averages and providing context for interpreting the trends. The difference in incidence between surveysin 2013 and 2016 is likely attributable to the high mortality seen in persons with epilepsy in onchocerciasis-endemic areas (1).

Table A – Gender-specific epilepsy prevalence in 2020 and 2022 in Mvolo County

Table A – Gender-specific epilepsy prevalence in 2020 and 2022 in Mvolo County, South Sudan.

**Prevalence**		**Survey**		**Significant difference** (95% CI_b)
		**2020***	**2022****	
**Confirmed lifetime epilepsy** N (prevalence per 1,000 persons, 95% CI_w)	**Male**	419/7,880 (53.2, 48.4–58.4)	339/7,390 (45.9, 41.3–51.0)	Yes (-1.4 – -0.1 pp)
	**Female**	378/7,863 (48.1, 43.5–53.1)	311/7,680 (40.5, 36.3–45.2)	Yes (-1.4 – -0.1 pp)
	**Significant difference** (Bootstrap 95% CI)	No (-1.2 – 0.2%)	No (-1.2 – 0.1%)	—

CI_b – Bootstrap confidence interval; N – Number; pp – Percentage points.
* In 2020, 12 epilepsy cases lacked gender information and were excluded from gender-specific calculations. Consequently, 6 individuals were subtracted from both the female and male denominators to adjust for this exclusion.
** In 2022, 22 epilepsy cases lacked gender information and were excluded from gender-specific calculations. Consequently, 11 individuals were subtracted from both the female and male denominators to adjust for this exclusion.

Table B — Obvious causes of epilepsy

Most persons with epilepsy met the criteria for onchocerciasis-associated epilepsy (Table S2). The reasons for not meeting the criteria remained consistent between surveys. However, there was a noticeable decrease in cases reporting severe malaria within five years prior to the onset of the first seizures, from 11.6% of persons with epilepsy in 2020 to 5.2% in 2022.

Table B – Persons with epilepsy in 2022 meeting the onchocerciasis-associated epilepsy criteria.

		**Persons with epilepsy**		**Significant difference** (95% CI_b)
		**2020** (732)	**2022** (622)	
**Obvious cause of epilepsy within five years before the first seizures onset** N (%)	**Severe malaria**	85 (11.6)	32 (5.2)	Yes (-9.4 – -3.6 pp)
	**Severe measles**	10 (1.4)	9 (1.5)	No (-1.2 – 1.4 pp)
	**Head injury with loss of consciousness**	8 (1.1)	4 (0.6)	No (-1.5 – 0.5 pp)
	**Encephalitis / meningitis / stroke**	5 (0.7)	2 (0.3)	No (-1.1 – 0.4 pp)
	**Other infection (tuberculosis or ear infection)**	2 (0.3)	0 (0.0)	No (-0.7 – 0.0 pp)
**New to the study area (<3 years of residence)** N (%)		11 (1.5)	7 (1.1)	No (-1.6 – 0.9 pp)
**First seizures onset**	**Before the age of 3 years** N (%)	34 (4.6)	26 (4.2)	No (-2-7 – 1.7 pp)
	**After the age of 18 years** N (%)	36 (4.9)	27 (4.3)	No (-2.8 – 1.6 pp)
**Meeting onchocerciasis-associated epilepsy criteria** N (%)		541 (73.9)	515 (82.8)	Yes (4.6 – 13.2 pp)

CI_b – Bootstrap confidence interval; N – Number; pp – Percentage points.

Table C – Longitudinal decrease in the incidence of overall epilepsy and probable nodding syndrome (NS) after reintroducing ivermectindistribution in Maridi and Mvolo Counties

There is a gradual, significant reduction in the incidence of overall epilepsy, encompassing probable NS, across both surveyed counties after the reintroduction of ivermectin distribution (Table S3). After nine years of ivermectin distribution, the incidence of probable NS has diminished to nearly negligible levels.

Table C – Changes in incidence rates of overall epilepsy and probable nodding syndrome (NS) from baseline following the reintroduction of ivermectin delivery.

County	Period	Overall Epilepsy		Probable NS	
		**Incidence** *n/100,000 (95% CI_w) PY*	**Difference from baseline** *pp (95% CI_b)*	**Incidence** *n/100,000 (95% CI_w) PY*	**Difference from baseline** *pp (95% CIb)*
**Mvolo**	2013–2015 (baseline)	326.5 (266.8–399.1)	–	151.7 (112.7-203.4)	–
	2015–2017	219.1 (170.8–280.5)	32.9 (9.1–51.4)	64.7 (40.6–101.9)	57.3 (29.5–76.1)
	2017–2019	136.7 (99.4–187.3)	58.1 (40.7–72.7)	74.5 (48.4–113.8)	50.9 (20.2–71.3)
	2019–2021	96.6 (65.5–141.7)	70.4 (56.4–81.7)	27.0 (12.5–55.5)	82.2 (66.0–93.5)
**Maridi***	2013–2017 (baseline)	360.7 (317.7–409.3)	–	157.2 (129.7–190.4)	–
	2017–2018	164.9 (111.5–242.2)	63.9 (47.2–78.2)	19.5 (5.5–58.6)	87.6 (72.1–100.0)
	2018–2020	163.2 (121.3–218.9)	54.3 (34.9–71.0)	38.2 (20.1–70.6)	75.7 (58.7–89.2)
	2020-2022	41.7 (22.6–75.0)	88.5 (81.1–94.6)	10.4 (2.7–33.2)	93.4 (84.7–100.0)

CI_b – Bootstrap confidence interval; CI_w – Wilson score confidence interval; N – Number; pp – Percentage points; PY – Person-years.

*Maridi data obtained from Jada SR, Amaral LJ, Lakwo T, Carter JY, Rovarini J, Bol YY, et al. Effect of onchocerciasis elimination measures on the incidence of epilepsy in Maridi, South Sudan: a 3-year longitudinal, prospective, population-based study. Lancet Glob Health. 2023;11(8):e1260-e8. doi: 10.1016/s2214-109x(23)00248-6. PubMed PMID: 37474232. Incidence values were recalculated by applying the rank weighting adjustment outlined in the referenced Maridi study while adhering to the same statistical approach employed for Mvolo in the present analysis to facilitate interpretation.

References

1. Siewe Fodjo JN, Van Cutsem G, Amaral LJ, Colebunders R. Mortality among persons with epilepsy in onchocerciasis-endemic and non-endemic areas of sub-Saharan Africa: A systematic review and meta-analysis. Seizure. 2023 Aug;110:253-261. doi: 10.1016/j.seizure.2023.07.006. Epub 2023 Jul 10. PMID: 37451075.
