# Supplementary material for: Impact of annual community-directed treatment with ivermectin on the incidence of epilepsy in Mvolo, a two-year prospective study
Source: PLoS Negl Trop Dis. 2024 Mar 21;18(3):e0012059. doi: 10.1371/journal.pntd.0012059 (PMC10986994; doi:10.1371/journal.pntd.0012059)
Supplement: Attachment — Submitted filename: Answers to the Reviewers Final.docx [file pntd.0012059.s008.docx]

**Revise Submission of “Impact of annual community-directed treatment with ivermectin on the incidence of epilepsy in Mvolo, a two-year prospective study”**

**Reviewer 1**

Q1.1) The methods need some more details and clarification. Page 3: What does the term, “reduced periods of consciousness” mean? Does it refer to duration of impairment of consciousness?

**Response**: The term "reduced periods of consciousness" refers to the episodes where there is a temporary impairment or alteration in consciousness. We clarified this in the manuscript: with periods of reduced consciousness. We appreciate the reviewer’s helpful insight.

Q1.2) Page 8, paragraph 1: The sentence refers to “five epilepsy screening questions”. But no details are provided. What were these questions?

**Response**: These questions were indeed mentioned earlier in the same paragraph. For comprehensive details, readers are directed to reference 40 and Supplementary Material S1, where these questions are fully described. To enhance clarity in our manuscript, we have now added the term 'aforementioned' before 'five epilepsy screening questions' to clearly indicate this reference.

Q1.3) Page 10: It is understandable that household surveys in this region of the world are challenging and particularly for making medical diagnoses in the field. But it would be useful to know how specific are the criteria being used for determining these clinical manifestations. For example, many exematous skin conditions could present with nodules, pruritis and scars. Similarly the assessment of cognitive function is limited to orientation. This would be considered highly inadequate for assessment of cognitive function. Nakalanga features are defined as thoracic or spine abnormalities with growth retardation. This also seems very non-specific for many medical conditions can cause such a phenoypye.

**Response**: We appreciate the reviewer’s observation regarding the limited scope of our cognitive function assessment. Indeed, our study specifically assessed 'Orientation' in terms of time, space, and person, as opposed to conducting a comprehensive evaluation of cognitive impairment. This targeted assessment was chosen due to the study's constraints and specific context. We recognise that this approach provides insights into only a limited aspect of cognitive function. Therefore, we have revised our manuscript to clearly state that our focus was on assessing ‘Orientation’.

It is indeed challenging to exhaustively exclude all potential etiologies of skin conditions in field settings. However, the presence of nodules, pruritic skin, leopard skin, scaly/thickened/wrinkled skin and severe itching in an onchocerciasis endemic area with high ongoing *O. volvulus* transmission is rather specific for onchocerciasis. Scabies is another diagnosis that needs to be suspected but scabies, as it is treatable with ivermectin is generally not very prevalent in areas where mass drug distribution of ivermectin is implemented.

Concerning the discussion of Nakalanga features, we acknowledge that a definite diagnosis of Nakalanga Syndrome is complex. In our study, the term “Nakalanga features” refers to specific observations, including visible growth retardation in individuals aged 16 years and above, often accompanied by thoracic or spinal abnormalities and the absence of external signs of secondary sexual development. These children are born without any noticeable abnormalities and develop typically. However, they later exhibit features often associated with the burden of an onchocerciasis infection. In the context of an onchocerciasis-endemic area, this constellation of features has historically been suggestive of possible Nakalanga Syndrome or a Nakalanga-like presentation. Hence, by using the term "Nakalanga features", we are specifically highlighting the observed characteristics while acknowledging the diagnostic uncertainty surrounding the syndrome. We have refined this definition in the methodology.

By mentioning “onchocerciasis-associated manifestations” instead of “onchocerciasis”, we aim to indicate that the clinical manifestations described are usually found in onchocerciasis-endemic settings.

Q1.4) Data analysis: No P values have been calculated for any of the observations. This is particularly important to substantiate any of the claim for decrease in the incidence or prevalence of NS or epilepsy in the population over time.

**Response**: In our cross-sectional study design, approximately 33% of the population of Mvolo was surveyed at two different time points, therefore, around a third of households were included in both surveys. This potential overlap challenges the assumption of independence between samples, which is a key requirement for the validity of p-values derived from Chi-squared tests. Therefore, we chose not to use the traditional p-values for comparison, as they might not accurately reflect the differences observed.

To address this issue, we employed bootstrap methods for our analysis. Bootstrap, a resampling technique that does not inherently assume the independence of observations and can be adjusted to account for the partial overlap, allowed us to generate more robust estimates of confidence intervals. We believe this approach provides a more reliable assessment of the changes in incidence and prevalence over time. We have updated our methodology for a detailed explanation of this methodology in the revised manuscript to ensure clarity and transparency in our analytical approach:

*“To account for the 33% overlap in surveyed populations in 2020 and 2022 (implying that the baseline and follow-up study populations were not completely independent), an adjusted Bootstrap method (producing 10,000 bootstrapped samples per comparison) was employed to calculate 95% confidence intervals bootstrap (CIb) to assess differences between surveys [50-52]. This non-parametric approach generated bootstrapped samples incorporating the specific overlap proportion, representing individuals that were surveyed both once and twice. For dichotomous variables, bootstrapping estimated and compared proportions with CIb For continuous variables, bootstrapped medians and their CIb were used to evaluate median differences. A sensitivity analysis was performed comparing the CIb with those obtained using the Wilson score method with continuity correction. Additionally, all 95% confidence intervals presented for single proportions and rates, including differences in ivermectin coverage, were determined using Wilson (w) Score Intervals (95% CIw) with Yate’s continuity correction for rare events [53]. A confidence interval that excluded the value of 0 was interpreted as statistically significant.”*

We also updated the discussion accordingly:

*“Moreover, the use of an adjusted bootstrap method to calculate confidence intervals, accounting for the 33% overlap found in households surveyed across the two years, further enhances this reliability. This adjustment acknowledges the potential non-independence of samples due to repeated household participation in successive surveys, providing a more realistic representation of our data. Additionally, a sensitivity analysis was conducted using the Wilson score interval with Yate's continuity correction on all presented bootstrap 95% confidence intervals to ensure the robustness of the bootstrap CIs to alternative methods and potential violations of normality assumptions. Consistent results between the confidence intervals obtained from the bootstrap method and Wilson score provided further confidence in the findings.”*

Q1.5) Page 15: What criteria were used to diagnose “onchocerca related blindness”? Does this population also have other causes of blindness such as vitamin A deficiency or trachoma etc.

**Response**: We removed the term "onchocerciasis-related blindness" and only reported blindness. *Onchocerca*-related blindness is usually assessed using portable slit lamp to detect the presence of microfilariae in the anterior chamber of the eye, but for this study this kind of diagnosis was not conducted.

Q1.6) Table 4: Please include dosage and frequency of administration of ivermectin.

**Response**: Ivermectin was administered according to the Mass Drug Administration (MDA) guidelines, which typically recommend a single oral dose of 150 to 200 micrograms per kilogram of body weight. In practice, however, the dosage is rather determined using body height instead of body weight. Children under the age of 5 years, pregnant women, women in the first week of lactation and individuals with severe disease are excluded from treatment. In our study, the MDA campaigns were conducted annually, consistent with the national guidelines for onchocerciasis control in South Sudan, which apply the WHO’s strategy of community-directed treatment with ivermectin (CDTI). The CDTI approach is already well documented and codified in literature in terms of dosages, eligibility, required treatment duration and coverage. We have added the months of CDTI delivery. We have added the months of CDTI delivery to the methodology as follows:

*“By 2016, South Sudan aimed to eliminate O. volvulus transmission through annual CDTI with 80% overall therapeutic coverage across all onchocerciasis-endemic regions [19], as per the 2016 World Health Organization guidelines [39]. Since then, annual CDTI was delivered in September 2017, August 2018, October 2019 and August 2021.”*

Q1.7) Table 5: How many patients with Nodding Syndrome also had other types of seizures? This could be an interesting population to study. It might be important to know if ivermectin had any impact of these different types of seizures in the same individual. Along the same lines, once NS has developed does ivermectin have any effect on the seizures?

**Response**: While the primary aim of our study was to determine the prevalence and incidence of epilepsy, including nodding syndrome, in the context of ivermectin intervention, we did not specifically measure the direct effect of ivermectin on the seizure patterns of those already diagnosed with NS. This remains a pertinent question for future research, as understanding the therapeutic effects of ivermectin on NS and concurrent seizure disorders could provide valuable insights into treatment strategies and the pathophysiology of the condition.

Regarding the co-occurrence of nodding syndrome and other types of seizures, we now provide information on the number of individuals that have probable nodding syndrome in co-occurrence with other types of seizures, termed “nodding syndrome plus”, on Table 5 as follows:

*“In 2022, 218 (80%) of the individuals with probable nodding syndrome had other types of seizures besides nodding seizures. Similarly, in 2020, 264 out of 342 individuals (77%) with probable nodding syndrome experienced other types of seizures. This co-occurrence of nodding and other seizures is denoted as nodding syndrome plus* *and is within the spectrum of onchocerciasis-associated epilepsy [8].”*

Q1.8) Table 5: It gives the frequency of seizures. But often they occur in clusters and not neatly in regular intervals of daily, weekly, etc. This information needs to be revised.

**Response**: We acknowledge the complexity of seizure patterns, particularly the occurrence of seizures in clusters, which may not align with the neat intervals of daily, weekly, etc., as initially represented in the table. To address this, we revised Table 5 to better reflect the variability and irregularity in seizure frequency by adding a row of “seizures experienced the week preceding the survey”. We also added to the results:

*“Almost two-thirds of the participants with epilepsy experienced at least one seizure episode in the week preceding the survey.”*

Q1.9) Page 26: Discussion: In the section on vector control strategies it would be useful to also discuss if besides humans if there are other animal reservoirs for onchocercha.

**Response**: Indeed, there are different species within the genus *Onchocerca*, each with its host preferences. However, *Onchocerca volvulus*, the species responsible for human onchocerciasis, is primarily an anthroponotic parasite with no significant animal reservoir. While other *Onchocerca* species do infect animals, they are generally not transmissible to humans (Ref 1). This specificity of *O. volvulus* to humans simplifies vector control strategies, focusing on interrupting transmission among human populations and controlling the blackfly vector populations nearby humans.

Reference 1: Cambra-Pellejà M, Gandasegui J, Balaña-Fouce R, Muñoz J, Martínez-Valladares M. Zoonotic Implications of Onchocerca Species on Human Health. Pathogens. 2020 Sep 17;9(9):761. doi: 10.3390/pathogens9090761. PMID: 32957647; PMCID: PMC7560048.

Q1.10) Discussion: It might be useful to speculate as to why partial protection with ivermectin continues to have a protective response against the development of NS.

**Response**: Indeed, this is an important discussion. We address it in the following discussion statement, which has been further refined: “*As a severe type of OAE, NS is believed to be associated with high O. volvulus microfilarial loads [11], which may be considerably suppressed with a suboptimal CDTI coverage of 60% [55,56]. This hypothesis is also supported by the rapid decline in the incidence of probable NS in both Mvolo and Maridi Counties following suboptimal ivermectin distribution (Supplementary Material S6) [47].*”

Further contributions to this discussion in the future could come from understanding the pathogenesis mechanism of nodding syndrome. While several hypotheses have been proposed (Ref 1), further research is essential to fully elucidate these mechanisms and to refine treatment strategies for optimal patient outcomes.

Reference 1: Hadermann A, Amaral LJ, Van Cutsem G, Siewe Fodjo JN, Colebunders R. Onchocerciasis-associated epilepsy: an update and future perspectives. Trends Parasitol. 2023 Feb;39(2):126-138. doi: 10.1016/j.pt.2022.11.010. Epub 2022 Dec 15. PMID: 36528471.

Q1.11) Are there other parasitic infections in the population that could be impacted by the use of ivermectin. Could they play a role in the clinical syndrome?

**Response**: Ivermectin is highly effective against a range of filarial infections, such as Lymphatic filariasis (LF) caused by *Wuchereria bancrofti*, (and *Brugia malayi* and *Brugia timori* in Asia*)*, which may exist in low-to-moderate prevalence in the study area (Ref 1). Additionally, it shows some efficacy against intestinal helminth infections like *Strongyloides stercoralis* and certain soil-transmitted helminths (STH) (Ref 2). STH might be present in low numbers in Mvolo (Ref 1). However, the role of these infections in the incidence and prevalence of epilepsy, including nodding syndrome, is not considered significant. Notably, ivermectin has been administered in areas where LF is prevalent without onchocerciasis, but a high prevalence of epilepsy, including nodding syndrome, is predominantly observed in regions with significant onchocerciasis (Ref 3). The presence of co-infections could potentially increase host tolerance to pathogens (Refs 3 and 4), thereby elevating the overall burden of infections and, consequently, possibly increasing the risk of developing neurological conditions like epilepsy.

Reference 1: South Sudan Ministry of Health. National Master Plan for Neglected Tropical Diseases 2016 – 2020 [cited 2023 30th of November]. Available from: <https://espen.afro.who.int/system/files/content/resources/SOUTH_SUDAN_NTD_Master_Plan_2016_2020.pdf>.

Reference 2: Hailu T, Nibret E, Amor A, Munshea A, Anegagrie M. Efficacy of Single Dose Ivermectin Against *Strongyloides stercoralis* Infection Among Primary School Children in Amhara National Regional State. Infect Dis (Auckl). 2020 Jun 15;13:1178633720932544. doi: 10.1177/1178633720932544. PMID: 32595279; PMCID: PMC7297470.

Reference 3: Hadermann A, Amaral LJ, Van Cutsem G, Siewe Fodjo JN, Colebunders R. Onchocerciasis-associated epilepsy: an update and future perspectives. Trends Parasitol. 2023 Feb;39(2):126-138. doi: 10.1016/j.pt.2022.11.010. Epub 2022 Dec 15. PMID: 36528471.

Reference 4: King IL, Li Y. Host-Parasite Interactions Promote Disease Tolerance to Intestinal Helminth Infection. Front Immunol. 2018 Sep 20;9:2128. doi: 10.3389/fimmu.2018.02128. PMID: 30298071; PMCID: PMC6160735.

Q1.12) It would be helpful to discuss the potential pathophysiological mechanisms by which *Onchocerca* infection might cause nodding syndrome. This would help strengthen their conclusions and observations.

**Response**: Thank you for your inquiry regarding the potential pathophysiological mechanisms underlying the association between onchocerciasis infection and nodding syndrome (NS). Several hypotheses have been proposed, but so far none has been confirmed yet, as highlighted in our recent publication (Ref 1) referenced in our paper. Research is underway is fully elucidate the pathophysiology of nodding syndrome to guide preventive and therapeutic decisions. In the meanwhile, it is believed to be associated with the number (burden) of microfilariae (onchocercal larvae) in the skin. Specifically, we discuss this association in the introduction, emphasising the significance of reducing the microfilarial load as a preventive measure against onchocerciasis-associated epilepsy (OAE):

*“Onchocerciasis-associated epilepsy has been particularly linked with O. volvulus microfilarial load, which denotes the number of microfilariae (the early larval stage of O. volvulus parasite) in the skin of the host [11]. As such, effective public health strategies aimed at preventing OAE should focus on decreasing the microfilarial load.”*

Furthermore, in the discussion section, we elaborate on how community-directed treatment with ivermectin (CDTI) likely impacts the microfilarial load and subsequently decreases the risk of developing OAE and NS. We provide evidence from our study context, including observations from Mvolo and Maridi Counties, to support these assertions:

Discussion section 1: “*As a severe type of OAE, NS is believed to be associated with high O. volvulus microfilarial loads [11], which may be considerably suppressed with a suboptimal CDTI coverage of 60% [55, 56]. This hypothesis is also supported by the rapid decline in the incidence of probable NS in both Mvolo and Maridi Counties following suboptimal ivermectin distribution (Supplementary Material S6) [47].*”

Additionally, we address the temporal dynamics of CDTI's impact on onchocerciasis burden, particularly among younger demographics who may have been excluded from treatment or received it at lower rates. We underscore the importance of sustained efforts in reducing microfilarial loads, especially in younger age groups and in highly endemic areas like Mvolo, to mitigate the risk of developing OAE and NS over time:

Discussion section 2: “*The reduction in epilepsy incidence became evident approximately a decade after the CDTI programme fully covered Mvolo. This delay aligns with the time required for ivermectin to effectively reduce the microfilarial load among the age group at risk for OAE, particularly in the context of Mvolo's suboptimal annual CDTI coverage and high baseline endemicity [26, 55-57]*”.

Discussion section 3: “*It is important to note that the younger age groups, either excluded from treatment until the age of five years or receiving it at lower rates (five to 19 years), were expected to hold considerable microfilarial loads [57]. Evidence of high ongoing onchocerciasis exposure among younger demographics in Mvolo was shown in 2020, with a substantial Ov16 seroprevalence among children under ten (27.3%, 95% CI: 20.8–34.9%) [41]. Consequently, a noticeable impact of CDTI on the onchocerciasis burden of younger demographics is expected to manifest several years after its initiation. This underscores the observed decrease in epilepsy incidence approximately a decade after the start of extensive CDTI. Since the risk of developing OAE is believed to be related to the skin microfilarial load of three- to 18-year-olds [11], the potentially pivotal role of ivermectin in reducing this load, especially in highly endemic areas like Mvolo, cannot be overstated*.”

Reference 1: Hadermann A, Amaral LJ, Van Cutsem G, Siewe Fodjo JN, Colebunders R. Onchocerciasis-associated epilepsy: an update and future perspectives. Trends Parasitol. 2023 Feb;39(2):126-138. doi: 10.1016/j.pt.2022.11.010. Epub 2022 Dec 15. PMID: 36528471.

Q1.13) This is an interesting study that shows that the use of an antiparasitic agent can have a dramatic effect of the incidence of epilepsy in a population with a high incidence of onchocerca infection. The observations are of public health importance and could have important socio-economic ramifications in these populations.

**Response**: Thank you for your insightful review and positive feedback.

**Reviewer 2**

Accept. Great work adding to the evidence of OAE.

Q2.1) Minor Revision; an explanation on why mosquito nets were considered in the data collection would provide clarity of the significance of bed net use in Onchocerciasis and OAE.

**Response**: Thank you for the positive and helpful review. In Mvolo, the consideration of mosquito nets in the data collection process was intended to provide clarity on the significance of bed net use in the context of onchocerciasis and onchocerciasis-associated epilepsy, particularly nodding syndrome (NS). In Mundri County in South Sudan, an association between nodding syndrome and onchocerciasis was documented, but also with *Mansonella perstans* infection (Ref 1). Given that bed nets can prevent malaria (a known risk factor for epilepsy) and could also confer some protection against *M. perstans* infection by stopping the biting midges (vectors of *M. perstans*) (Ref 2), we sought to investigate whether bed net use was a determinant for epilepsy/NS in our study. The bed net variable was found to be impractical for data analysis due to the low usage of bed nets among the population in Mvolo. Consequently, we have removed the bed net information from the study..

Reference 1: Jada SR, Siewe Fodjo JN, Abd-Elfarag G, Tionga M, Carter JY, Logora MY, et al. Epilepsy-related stigma and cost in two onchocerciasis-endemic areas in South Sudan: A pilot descriptive study. Seizure. 2020;81:151–6. pmid:32810838

Reference 2: Baker T, Carpenter S, Gubbins S, Newton R, Lo Iacono G, Wood J, et al. Can insecticide-treated netting provide protection for Equids from Culicoides biting midges in the United Kingdom? Parasit Vectors. 2015;8:604. pmid:26607993

**Reviewer 3**

Q3.1) The study design is strengthened by the clinically-confirmed diagnosis of epilepsy, including pNS. The study design was also clear, being described as two cross-sectional surveys.

Page 8: The methodology is incomplete, as there is no description of the ivermectin regimen, such as what months the participants took the treatment or the specific dosage of treatment. Additionally, no details are provided on when the surveys were conducted after treatment. How many weeks or months after ivermectin intake were the participants surveyed? If it was long after, there could be an issue of recall bias.

**Response**: Thank you for your valuable feedback regarding the methodology section of our study. We have now updated the methodology to include details on the ivermectin regimen:

“*By 2016, South Sudan aimed to eliminate O. volvulus transmission through annual CDTI with 80% overall therapeutic coverage across all onchocerciasis-endemic regions [19], as per the 2016 World Health Organization guidelines [39]. Since then, annual CDTI was delivered in September 2017, August 2018, October 2019 and August 2021.*”

The 2020 and 2022 surveys were conducted eight and ten months after the last treatment round, respectively. We acknowledge that while there is a possibility of recall bias, the high endemicity of onchocerciasis in Mvolo and the community's awareness of the condition (targeted by an awareness campaign in 2020) and the need for preventive treatment may help mitigate this bias to some extent Ref 1. We have updated the discussion accordingly:

“*However, there remains a possibility of recall bias when retrospectively calculating the epilepsy incidence and ivermectin coverage. The former was mitigated by calculating the incidence every two years. Concerning the latter, while there is a potential for recall bias as coverage data were assessed eight and ten months after the last treatment round in 2019 and 2021, respectively, the high endemicity of onchocerciasis in Mvolo, the community's awareness of the condition, which was targeted by an awareness campaign in 2020 [41], and the need for preventive treatment may help minimise this bias. Moreover, directly asking the population about their intake of ivermectin provides additional insights besides the coverage reported by the community drug distributors,* *such as accounting for individuals who received ivermectin but opted not to take it take it (e.g. reason for non-adherence) [49, 73, 74], and households* *inadvertently overlooked during the CDTI rounds*.”

Q3.2) Page 10, paragraph 1: Onchocerciasis-related blindness was clinically defined, but similar blindness can be caused by trachoma, a public health issue also endemic in South Sudan, and vitamin A deficiency. Thus, there is a caveat to this definition, and the limitation of the clinical diagnosis should be discussed later in the manuscript as well.

**Response**: We dropped the term "onchocerciasis-related blindness" and only kept the term blindness.

Q3.3) Page 10, paragraph 1: Cognitive impairment was defined as "when an individual is not fully oriented in space, time, and person." However, this symptom can be caused by a number of things that are not included in the manuscript's research. Therefore, this variable is not well defined in relation to onchocerciasis.

**Response**: Indeed, we assess cognitive impairment (disorientation) in the scope of epilepsy, rather than onchocerciasis. Our aim with this variable is to understand the cognitive impairment of persons with epilepsy in South Sudan and understand if providing antiseizure medication could alleviate it. Therefore, we could use this variable to draw a brief comparison between Mvolo and Maridi Counties, adjacent counties where in the former most persons with epilepsy do not have access to antiseizure medication, while in the latter antiseizure medication is delivered free of charge. Before the delivery of antiseizure medication, both counties had comparable proportion of cognitive impairment (disorientation), whereas afterwards it significantly decreased in Maridi, while slightly increasing in Mvolo. We have updated the discussion accordingly:

“While the prevalence of epilepsy in Mvolo remains high at 4–5%, there was a notable reduction in prevalence among those under 20 years of age by 11.4 (95% CI: 6.1–16.9) percentage points from 2020 to 2022. This reduction supports the observed decline in epilepsy incidence, which in endemic areas is predicted to predominantly occur in children and adolescents when onchocerciasis elimination measures are introduced or strengthened [15]. A similar result was described in Maridi County, with a decrease in epilepsy prevalence of 16.4 (95% CI: 11.3–21.5%) percentage points for the same age group between 2018 and 2022 [14]. In contrast, epilepsy prevalence in individuals aged over 20 years remained unchanged in Mvolo, while Maridi saw a significant increase of 16.3%. Another relevant metric is the proportion of persons with epilepsy “not oriented in time, space and person”, which slightly increased in Mvolo (from 14.7% to 20.5%), while significantly decreasing in Maridi (from 21.3% to 9.7%). This difference is likely explained by Maridi’s longer study duration and lower epilepsy-related mortality [58] due to the establishment of epilepsy clinics in 2020, where antiseizure medication is provided free of charge [59]. Whereas the reported uptake of antiseizure medications significantly increased in Maridi from 51.4% in 2018 to 91.1% in 2022, it was persistently low in Mvolo (~20%) in 2020 and 2022. These findings emphasise the need to decrease the epilepsy treatment gap in Mvolo and across sub-Saharan Africa [60, 61].”

Q3.4) Page 10, paragraph 2: The data analysis involved a “bootstrapping method” and the “Bootstrap 95% Confidence Interval.” Given that this is an uncommonly used methodology, it should be at least referenced by an outside source in this section.

**Response**: Indeed, we have updated the methodology accordingly:

*“To account for the 33% overlap in surveyed populations in 2020 and 2022 (implying that the baseline and follow-up study populations were not completely independent), an adjusted Bootstrap method (producing 10,000 bootstrapped samples per comparison) was employed to calculate 95% confidence intervals bootstrap (CIb) to assess differences between surveys [50-52]. This non-parametric approach generated bootstrapped samples incorporating the specific overlap proportion, representing individuals that were surveyed both once and twice. For dichotomous variables, bootstrapping estimated and compared proportions with CIb. For continuous variables, bootstrapped medians and their CIb were used to evaluate median differences. A sensitivity analysis was performed comparing the CIb with those obtained using the Wilson score method with continuity correction.”*

Q3.5) All of the results were presented in tables and the abbreviations were explained below the relevant tables. Data analysis: Again, how does the Bootstrap 95% Confidence Interval relate to statistical significance? How can one tell the confidence interval is statistically significant without the calculation of p values?

**Response**: As described in the methodology, a confidence interval not including the value of 0 (e.g. 95%CI: 6.3 – 19.5) is considered statistically significant (p < 0.05). Methodology:

“*A confidence interval that excluded the value of 0 was interpreted as statistically significant.”*

Q3.6) Page 12, paragraph 1; page 16, paragraph 1: The terms "percent" and "percentage points" are used interchangeably when referring to a change in the data points for the two surveys. This is incorrect, as only percentage points refers to the difference between percentages.

**Response**: We thank the reviewer for the remark. The manuscript has been updated accordingly to describe “percentage points” where applicable.

Q3.7) There are some limitations missing from the discussion section. While the implementation of CDTI was concluded to be followed by a reduction in epilepsy, it is not discussed why the results section included data (table 8) showing epilepsy on the decline since 2013. Additionally, the manuscript infers implementation of ivermectin only from 2019-2021. Thus, is there a possibility the decrease between 2020-2022 is due to the continuation of a previous trend, confounding variables, or other standard of care measures rather than ivermectin? This limitation regarding ivermectin-related reduction in epilepsy is especially concerning given the suboptimal coverage by the second survey. Or, can the author confirm and specify if rounds of ivermectin treatment were distributed from 2013 onwards, possibly contributing to the trend of decreased epilepsy incidence.

**Response**: The data from July 2013 to July 2015 were included as a baseline to illustrate the continuous decline in epilepsy incidence. We refrained from extending the retrospective analysis further back to prevent recall bias, as mentioned in the discussion (manuscript section 1 below). Ivermectin, as detailed in the methodology, has been delivered in Mvolo since the early 2000s, initially with very low coverage (manuscript section 2). Ivermectin only kills the progeny of the worm (microfilariae) and not the adult worms (manuscript section 3 below). Hence, it needs to be given to all individuals at risk for at least 10 to 15 years, representing the lifespan of the adult worm of *O. volvulus* (manuscript section 4 below). However, in areas as endemic as our study area and with suboptimal coverage, the treatment will be needed for more time, and take longer to reduce the number of microfilariae in the skin, which is believed to be related to the risk of developing epilepsy. We acknowledge the need for a more detailed discussion of the relationship between ivermectin delivery and epilepsy incidence, and we added:

*“The observed decline in epilepsy incidence in Mvolo began around 2016, as illustrated in Figure S1. This downward trend aligns with the phased implementation of the CDTI programme, initiated in the early 2000s. While the programme initially achieved very low coverage, it progressively expanded over time, albeit remaining below the optimal threshold recommended by the World Health Organization for the elimination of onchocerciasis transmission [39]. The reduction in epilepsy incidence became evident approximately a decade after the CDTI programme fully covered Mvolo. This delay aligns with the time required for ivermectin to effectively reduce the microfilarial load among the age group at risk for OAE, particularly in the context of Mvolo's suboptimal annual CDTI coverage and high baseline endemicity [26, 55-57].*

*Moreover, the suppression of microfilariae production in the adult population, which largely contributes to the transmission of O. volvulus, indirectly reduces the exposure of young children to onchocerciasis. It is important to note that the younger age groups, either excluded from treatment until the age of five years or receiving it at lower rates (five to 19 years), were expected to hold considerable microfilarial loads [57]. Evidence of high ongoing onchocerciasis exposure among younger demographics in Mvolo was shown in 2020, with a substantial Ov16 seroprevalence among children under ten (27.3%, 95% CI: 20.8–34.9%) [41]. Consequently, a noticeable impact of CDTI on the onchocerciasis burden of younger demographics is expected to manifest several years after its initiation. This underscores the observed decrease in epilepsy incidence approximately a decade after the start of extensive CDTI. Since the risk of developing OAE is believed to be related to the skin microfilarial load of three- to 18-year-olds [11], the potentially pivotal role of ivermectin in reducing this load, especially in highly endemic areas like Mvolo, cannot be overstated.”*

Manuscript sections:

1) “*These periods were chosen to observe the temporal trends of epilepsy incidence while taking into consideration the potential for recall bias and the possibility of missing newly developed epilepsy cases* (Supplementary Material S3).”

2) “*The CDTI programme in the county started in 2002, initially targeting a mere 12.4% of at-risk communities [28, 29]. In 2006, the African Programme for Onchocerciasis Control (APOC) extended annual CDTI to the remaining communities [30], and CDTI has been sustained ever since except in 2016 and 2020 (the latter due to COVID-19 restrictions) [24, 30-38]. By 2016, South Sudan aimed to eliminate O. volvulus transmission through annual CDTI with 80% overall therapeutic coverage across all onchocerciasis-endemic regions [19], as per the 2016 World Health Organization guidelines [39]. Since then, annual CDTI was delivered in September 2017, August 2018, October 2019 and August 2021*.”

3) “*Ivermectin is a microfilaricidal drug that eliminates microfilariae and temporarily sterilises female adult worms* [13].”

4) “*This slower change is attributed to the ability of adult worms to produce microfilariae for over a decade, even in the absence of ongoing transmission [16], and the density-dependent processes that govern the lifecycle of the parasite* [17].”

Q3.8) Page 25, paragraph 1: It would be useful to emphasize the importance of not only strengthening access to ivermectin, but also increasing treatment coverage specifically towards the younger, more at risk age group. This is especially important as the younger age group had decreased CDTI compared to the older participant group.

**Response**: We fully agree with the reviewer's assessment. Strengthening access to ivermectin and increasing treatment coverage, especially among younger age groups, is paramount. We mentioned the need to increase coverage among younger age groups through the discussion and we have now highlighted this point in the updated discussion segment provided in response to the reviewer's previous question. Thank you for bringing attention to this crucial aspect.

Q3.9) This study is important in emphasizing a relation between onchocerciasis and epilepsy, as epilepsy incidence may also improve with the use of ivermectin in endemic regions. This link is important for the public health field, as increased CDTI could lead to health benefits among these populations. However, there needs to be some more thought to the limitation section, specifically regarding the previously declining epilepsy trend.

**Response**: Thank you for your valuable review and feedback. The previously declining epilepsy trend aligns with our expectations for Mvolo, where ivermectin delivery began in the early 2000s with initially low coverage, gradually increasing yet remaining below the recommended threshold in WHO guidelines for onchocerciasis elimination. In the methodology:

*“The CDTI programme in the county started in 2002, initially targeting a mere 12.4% of at-risk communities [28, 29]. In 2006, the African Programme for Onchocerciasis Control (APOC) extended annual CDTI to the remaining communities [30], and CDTI has been sustained ever since except in 2016 and 2020 (the latter due to COVID-19 restrictions) [24, 30-38]. By 2016, South Sudan aimed to eliminate O. volvulus transmission through annual CDTI with 80% overall therapeutic coverage across all onchocerciasis-endemic regions [19], as per the 2016 World Health Organization guidelines [39]. Since then, annual CDTI was delivered in September 2017, August 2018, October 2019 and August 2021.”*

As depicted in Figure S1, the epilepsy incidence in Mvolo shows a notable decrease from 2016, a decade after the expansion of CDTI. This timeframe corresponds with the gradual reduction in microfilarial load, especially in younger age groups who are either excluded from ivermectin treatment (until the age of five years) or have lower ivermectin coverage (as shown in Table 7). The delayed impact on epilepsy incidence is consistent with the time needed for ivermectin to reduce microfilariae intensity and transmission and subsequently lower the exposure of younger children to onchocerciasis (Refs 1 and 2). Therefore, the observed trends are in line with the expected gradual effects of sustained, albeit suboptimal, ivermectin administration in a highly endemic area. We have added this explanation to the discussion as follows:

*“The observed decline in epilepsy incidence in Mvolo began around 2016, as illustrated in Figure S1. This downward trend aligns with the phased implementation of the CDTI programme, initiated in the early 2000s. While the programme initially achieved very low coverage, it progressively expanded over time, albeit remaining below the optimal threshold recommended by the World Health Organization for the elimination of onchocerciasis transmission [39]. The reduction in epilepsy incidence became evident approximately a decade after the CDTI programme fully covered Mvolo. This delay aligns with the time required for ivermectin to effectively reduce the microfilarial load among the age group at risk for OAE, particularly in the context of Mvolo's suboptimal annual CDTI coverage and high baseline endemicity [26, 55-57].*

*Moreover, the suppression of microfilariae production in the adult population, which largely contributes to the transmission of O. volvulus, indirectly reduces the exposure of young children to onchocerciasis. It is important to note that the younger age groups, either excluded from treatment until the age of five years or receiving it at lower rates (five to 19 years), were expected to hold considerable microfilarial loads [57]. Evidence of high ongoing onchocerciasis exposure among younger demographics in Mvolo was shown in 2020, with a substantial Ov16 seroprevalence among children under ten (27.3%, 95% CI: 20.8–34.9%) [41]. Consequently, a noticeable impact of CDTI on the onchocerciasis burden of younger demographics is expected to manifest several years after its initiation. This underscores the observed decrease in epilepsy incidence approximately a decade after the start of extensive CDTI. Since the risk of developing OAE is believed to be related to the skin microfilarial load of three- to 18-year-olds [11], the potentially pivotal role of ivermectin in reducing this load, especially in highly endemic areas like Mvolo, cannot be overstated.”*
